# Exploring Familial Adenomatous Polyposis Through Radiology: A Case Series and Literature Review

**DOI:** 10.7759/cureus.64855

**Published:** 2024-07-18

**Authors:** Seetha Rashi, Abdul Majith Seeni Mohamed, Karthik Krishna Ramakrishnan, Sakthi Ganesh Subramonian, Vadupu Udaya Bhanu

**Affiliations:** 1 Department of Radiology, Saveetha Medical College and Hospital, Saveetha Institute of Medical and Technical Sciences, Saveetha University, Chennai, IND

**Keywords:** colorectal cancer, autosomal dominant, gastrointestinal symptoms, familial adenomatous polyposis, apc gene

## Abstract

Familial adenomatous polyposis (FAP) is a dominantly inherited, autosomal form of hereditary condition caused by a germline mutation in the adenomatous polyposis coli (APC) gene. The early development of adenomatous polyps in the colon and rectum predisposes to rampant proliferation, which usually leads to colorectal cancer. Hence, this condition demands intensive surveillance and aggressive intervention. This case report epitomizes the convergence of advanced imaging with genetic diagnosis and, in essence, points toward a complete multidisciplinary approach as critical for proper management of FAP. The detailed evaluation of two siblings presenting with similar gut symptoms from this article focused on the individualization that this condition needs when managed, although underpinning the critical role coordinated care plays in changing disease outcomes.

## Introduction

Familial adenomatous polyposis (FAP) is one of the major challenges in the field of colorectal cancer syndromes. This is an autosomal dominant condition, though very rare, that is clinically relevant as it predisposes to the formation of multiple adenomatous polyps throughout the colon and rectum, usually in early adulthood [1]. This is a clear illustration of how genetic susceptibility can result in a cascade of oncogenic changes, mostly resulting from the mutation of the adenomatous polyposis coli (APC) gene [2]. In this regard, such patients are prone to developing colorectal cancer as polyps keep on growing and therefore need appropriate monitoring together with treatment [3]. The clinical presentation for FAP ranges from chronic gastrointestinal (GI) symptoms to, at times, acute complications that point towards FAP in its advanced stages. Signs like persistent diarrhea, blood in the stools, and abdominal pain are often the initial signs of FAP; however, more often, the diagnosis is cloudy due to the absence of clinical signs [4]. In this regard, we have described this case series, which we hope will help in expounding on the complexity of the process of diagnosing and managing FAP. With contrast-enhanced computed tomography (CECT), we detail the polypoidal lesions, which provides a general overview of the effects of the disease. This is a comprehensive overview of familial adenomatous polyposis (FAP) by integrating clinical, radiological, and genetic data to improve the management strategies for hereditary colorectal cancer syndromes.

## Case presentation

**Figure 1:**
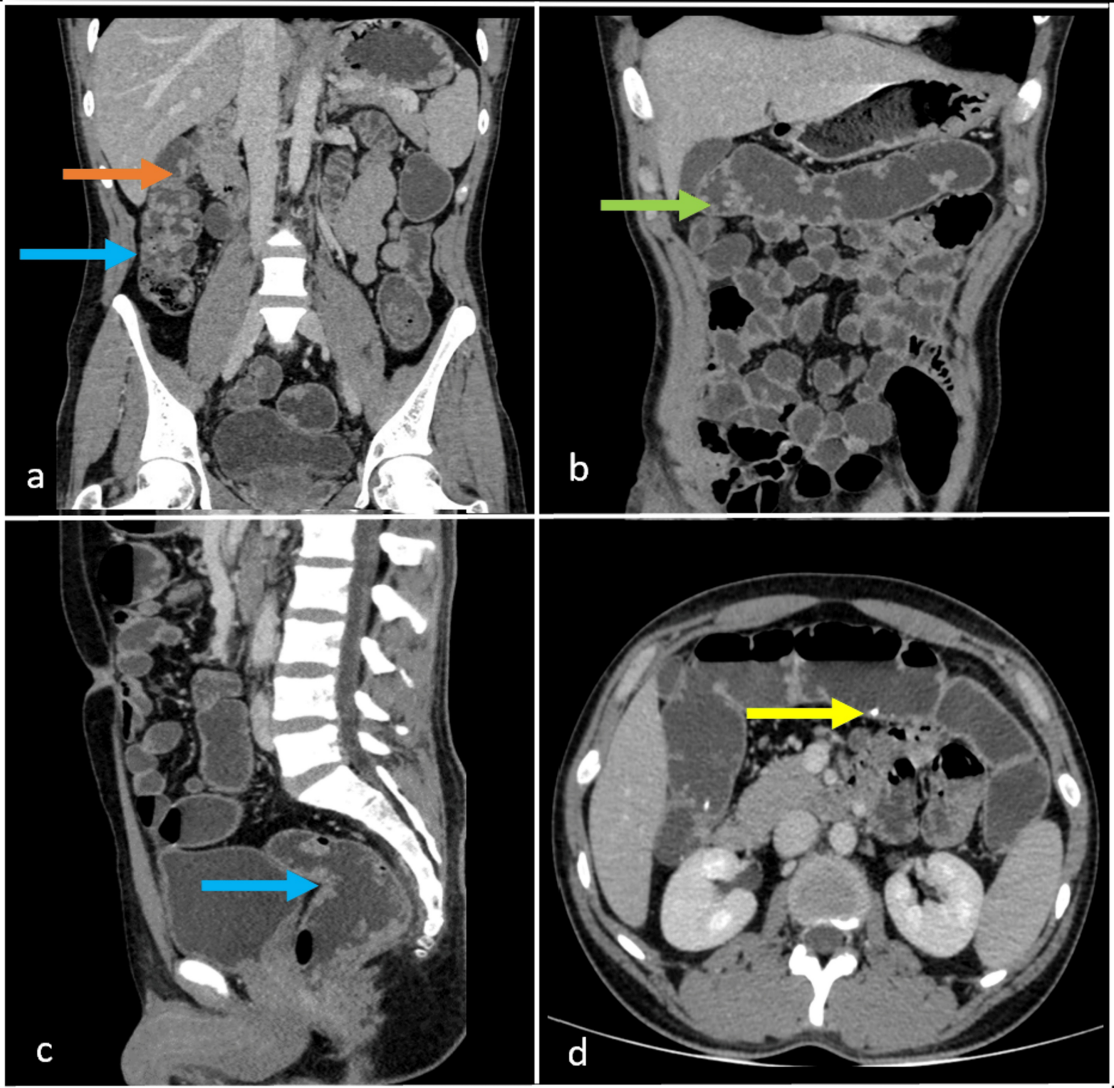
Contrast-enhanced CT abdomen coronal (a and b), sagittal (c), and axial (d) sections of the venous phase show multiple soft tissue polypoidal lesions (blue arrow), both sessile (green arrow) and pedunculated (orange arrow), and a few of them show calcifications (yellow arrow) noted involving the entire large bowel, which on post-contrast shows homogenous enhancement.

**Figure 2:**
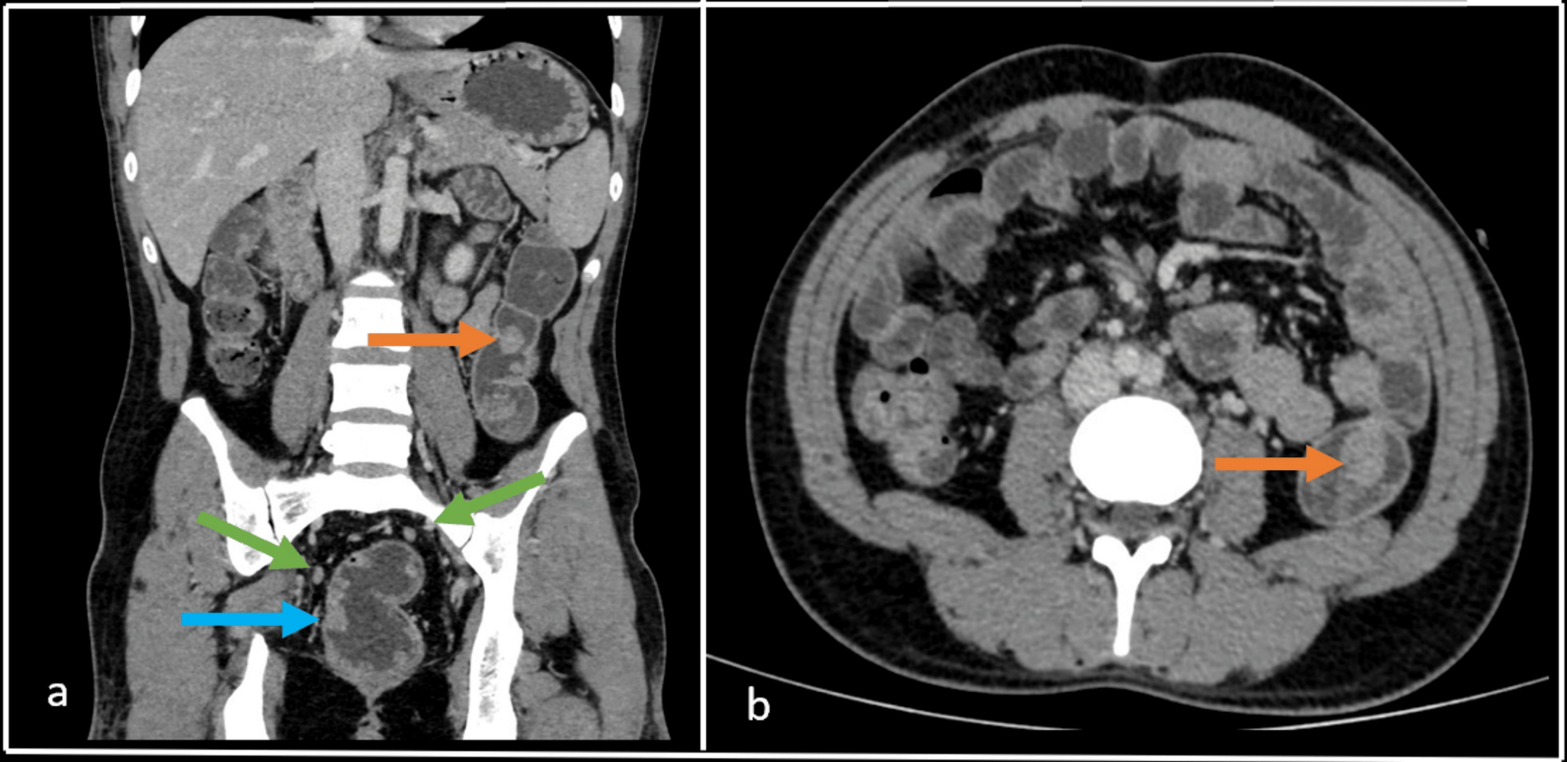
Contrast-enhanced CT abdomen coronal (a) and axial (b) venous phase shows the largest of the polyps in the rectum (blue arrow) and descending colon (orange arrow) measuring ~ 22 x 22 mm and 18 x 18 mm, respectively. Few homogenously enhancing sub-centimetric nodes were seen in mesorectum nodes (green arrow).

Sibling A, a 34-year-old well-nourished male with stable vitals, came to the outpatient department (OPD) with complaints of persistent gastroenteritis and altered bowel habits over the past few months. There was no significant past medical history. Abdominal examination revealed mild tenderness on palpation; however, there was no rebound tenderness or palpable masses. All the baseline investigations were within normal limits, hence inconclusive, and the patient has proceeded with CECT abdomen. The CECT abdomen revealed multiple sessile and pedunculated soft tissue polypoidal lesions, a few of which showed calcifications throughout the entire large bowel, involving the rectum, sigmoid colon, ascending, descending, transverse colon, and caecum. The largest of the polyps was in the rectum and descending colon, measuring ~ 22 x 22 mm and 18 x 18 mm, respectively. Post-contrast, the polyps showed homogenous enhancement. There was no evidence of transmural extension peri-focal fat stranding. Few homogenously enhancing sub-centimetric nodes were seen in the mesorectum and mesocolon lymph node stations, the largest of them measuring ~ 7 x 6 mm in the mesorectum (Figure 1, 2).

**Figure 3:**
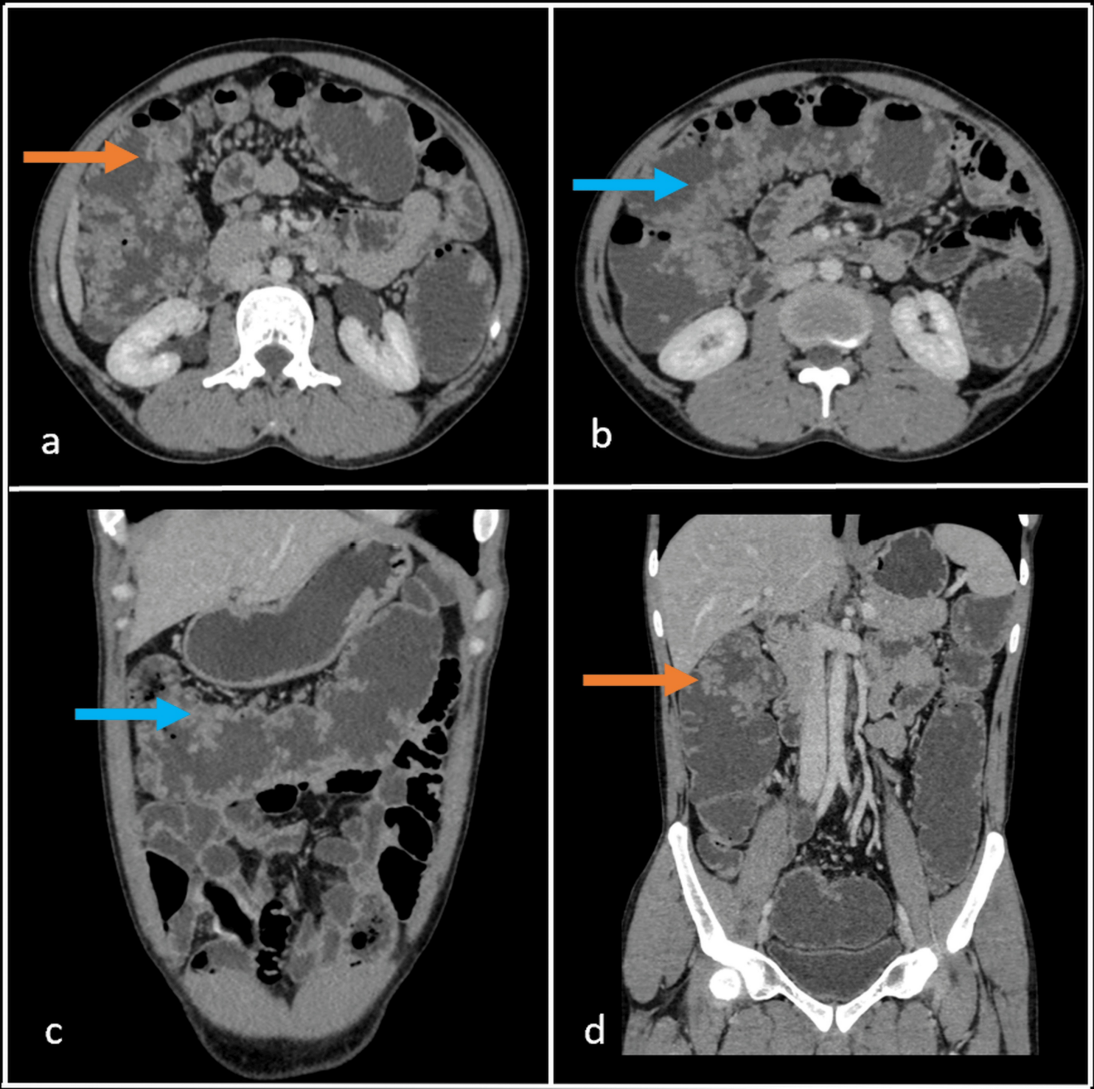
Contrast-enhanced computed tomography (CT) abdomen axial (a, b) and coronal (c, d) sections venous phase showing homogenously enhanced multiple soft tissue polypoidal lesions spanning the entire colon- ascending (orange arrow), transverse, and descending colons, with maximum involvement seen in the transverse colon (blue arrow).

**Figure 4:**
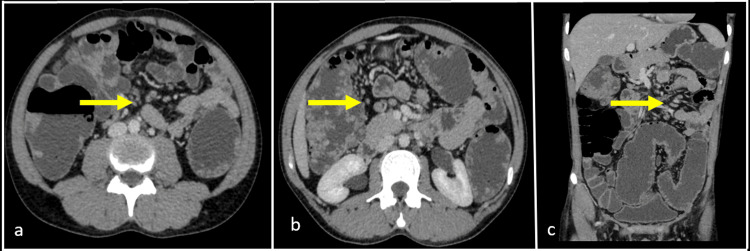
Contrast-enhanced computed tomography (CT) abdomen axial (a, b) and coronal (c) sections venous phase shows multiple prominent homogenously enhancing meso-colic nodes seen (yellow arrow).

Following concerns that Sibling A might be affected by familial adenomatous polyposis, raising the possibility of a shared genetic predisposition, Sibling B was requested to undergo diagnostic testing. Sibling B, experiencing gastrointestinal complaints such as abdominal discomfort, generalized fatigue, and occasional rectal bleeding similar to Sibling A, was advised to undergo further evaluation. Sibling B was of normal build, well-nourished, and had stable vitals. Per abdomen examination showed no palpable masses or abdominal tenderness, and all baseline investigations were within normal limits. A CECT abdomen was performed, revealing multiple soft tissue polypoidal lesions originating from the inner wall of the ascending colon, spanning the entire transverse, descending, and sigmoid colon, and extending to the distal anorectal region, with a predominance in the transverse colon, and the largest of them measuring ~10 x 10 mm. On post-contrast, the polyps showed mild enhancement (Figure 3). Additionally, prominent enhancing mesocolic and mesorectal nodes were noted, depicting regional lymphadenopathy (Figure 4).

Following the identification of compelling features indicative of familial adenomatous polyposis (FAP) in both siblings, APC genetic testing was pursued and yielded positive results. Consequently, the patients were strongly recommended to undergo surgical interventions: Sibling A was advised to have a proctocolectomy with J-pouch formation and ileostomy, while Sibling B was advised to have a prophylactic colectomy with ileal pouch-anal anastomosis.

## Discussion

Familial adenomatous polyposis (FAP) represents a significant hereditary colorectal cancer syndrome [1]. This autosomal dominant disorder is primarily caused by germline mutations in the adenomatous polyposis coli (APC) gene, leading to the development of numerous adenomatous polyps throughout the colon and rectum. These polyps continue to grow and increase the risk of colorectal cancer to a great extent, which underlines the necessity of early diagnosis of the disease. It is estimated that if FAP patients do not opt for prophylactic surgery before the age of forty, they are almost certain to develop colorectal cancer (CRC) [2,3].

In the case of Sibling A and Sibling B with multiple polypoidal lesions at the colon, the diagnosis of which was made by genetic testing of the APC gene, FAP, this paper underlines the necessity for the early and adequate investigation of the suspected hereditary CRC [1,2]. PCR amplification and sequencing of the gene established the mutation of the APC gene, which supports the previous findings of FAP hereditary disposition [2].

Pathophysiology and genetics

FAP is an autosomal dominant disorder, and the main gene involved in its development has been identified as adenomatous polyposis coli (APC) on chromosome 5q21 [2,3]. This mutation, which is inherited as an autosomal dominant trait, leads to the widespread and rapid division of the colonic epithelial cells that form multiple adenomatous polyps in the colon and rectum; these are premalignant lesions with a very high risk if not resected of future transformation into colorectal cancer.

Clinical presentation: gastrointestinal manifestations

Adenomatous Polyps

The hallmark of familial adenomatous polyposis (FAP) is the early onset, typically during adolescence, of hundreds to thousands of adenomatous polyps throughout the colon and rectum. Initially asymptomatic, the increase in polyp burden eventually leads to symptoms such as rectal bleeding, abdominal pain, and a change in bowel habits. In addition to colorectal polyps, patients with FAP commonly develop polyps in the upper gastrointestinal tract, particularly in the stomach and duodenum, with a significant risk of these polyps progressing to cancer, especially in the duodenum [2,4]. Without prophylactic surgery, virtually all individuals with FAP will develop colorectal cancer, usually by the age of 40. The risk of cancer correlates with the number of polyps and the duration for which they are present [3].

The presence of numerous sessile and pedunculated polyps across the entire colon is indicative of the typical FAP presentation. The largest of the polyps measured approximately 22 x 22 mm in the rectum, 18 x 18 mm in the descending colon in Sibling A, and ~10 x 10 mm in Sibling B, which is consistent with the size range observed in advanced polyp cases. Homogenous enhancement of these polyps on post-contrast imaging is a characteristic feature of FAP-associated polyps, as described in the literature.

Extra-colonic Manifestations

Desmoid tumors, benign yet potentially aggressive fibrous tumors, can arise from the mesentery or abdominal wall, causing pain and complications due to their mass effect on abdominal organs [5-7]. Osteomas, bony growths, particularly in the jaw and skull, are usually asymptomatic but can lead to cosmetic concerns or dental issues. Congenital hypertrophy of the retinal pigment epithelium (CHRPE) presents as pigmented lesions in the retina, which typically do not affect vision but serve as a marker for FAP [8]. Additionally, there is an increased incidence of thyroid cancer in individuals with FAP, particularly young women. The APC gene mutation responsible for FAP also contributes to the development of thyroid cancer by affecting cell growth regulation, leading to the formation of polyps in the colon and malignancies in other tissues, including the thyroid [9].

FAP increases the likelihood of developing colorectal cancer, as well as linked phenotypes such as desmoid tumors and adenomas in the upper GI tract [6,7]. The genetic counseling of both children and the following positive APC tests in two siblings highlight an essential role for imposed-sequencing/genetic diagnosing, cueing up maximum-risk families to primordial prevention and surveillance opportunities [2]. Also, patients with FAP have an increased risk of other malignancies, including thyroid and pancreatic carcinoma; thus, this group of patients requires a multidisciplinary approach to management and long-term follow-up [9]. Traboulsi et al. described the occurrence of pigmented ocular fundus lesions in patients with Gardner’s syndrome, one of the FAP subtypes, thus expanding the list of potential complications [8].

Other Associated Conditions

Hepatoblastoma, a variant of primary liver cancer, occurs in children with FAP. Hepatoblastoma screening in these children is advised based on the following schedule: abdominal ultrasound and serum alpha-fetoprotein (AFP) every three months for the first year, every two months for the second year, and every three months thereafter. Hepatoblastoma should be diagnosed and treated as early as possible, and this can be achieved through regular screening, which enhances the prognosis [5]. Also, it has been suggested that FAP patients might have an increased prevalence of benign tumors of the adrenal gland, which are often asymptomatic.

The diagnosis of FAP is usually clinical and is made if the patient has more than 100 colorectal adenomas. Molecular diagnosis is accomplished by the identification of specific mutations in the APC gene, which is also used for screening family members. Flexible endoscopy of the colon and rectum, usually colonoscopy, is important in diagnosing FAP and determining the extent of polyposis [10,11].

Radiologic features

Abdominal contrast-enhanced computed tomography (CECT) is not the initial diagnostic modality for FAP; however, it gives significant information about the severity and complications of the disease (the gold standard for screening FAP is colonoscopy) [10]. On imaging, the characteristic feature of FAP is the presence of numerous polyps within the colon and rectum, which may appear as multiple small nodular protrusions into the bowel lumen, and the colon may appear thickened.

In the cases of Sibling A and Sibling B, CECT of the abdomen demonstrated typical characteristics of FAP, such as multiple sessile and pedunculated polyps covering the mucosa of the colon and rectum. These polyps looked like multiple small nodular formations that grew into the lumen of the bowel and gave the colon a thickened look. This imaging finding corresponds to the descriptions in the literature, as the absence of polyps limited to one segment of the colon is characteristic of FAP [1,2,10].

The imaging findings of both siblings are in accordance with the literature, where CECT has been shown to detect multiple polyps in the colon and rectum in FAP patients [3,4]. There is another interesting feature of Sibling A’s polyps: while some of them are small, others are larger and contain calcifications; this represents the disease process and polyp maturation, which have also been described in the literature [6]. The presence of sub-centimetric nodes in the mesorectum and mesocolon also highlights the need for a thorough radiologic evaluation in FAP, as it is an early sign of disease progression or metastasis [5].

Further, FAP patients are at risk of desmoid tumors; although these are benign, they are aggressive fibrous neoplasms that may occur in the abdomen or pelvis. These tumors may appear on CECT as well-defined or infiltrative soft tissue masses that enhance the contrast and may be complicated by the compression of adjacent structures and by bowel obstruction [10,11].

Apart from desmoid tumors, FAP patients may develop other tumors, which may be adenocarcinomas in the colorectal area and may present as irregular, enhancing masses with local invasion of surrounding structures. Lymphadenopathy in the region could be suggestive of metastasis, particularly if the situation has resulted in colorectal adenocarcinoma. Free fluid in the abdominal cavity might be seen in advanced cases or complications such as perforation, infection, or advanced disease. FAP patients are also prone to developing other complications in the body, such as liver lesions in the form of hepatoblastoma or hepatic adenomas, which present as focal liver lesions with different enhancement patterns in CECT. Other organ lesions, such as those involving the pancreas and thyroid, should also be looked for, and Gardner’s syndrome, a variant of FAP that may present with osteomas and other bone lesions, may be an incidental finding noted on imaging that includes the pelvis or spine [12].

Occasionally, vascular pathologies, including aneurysms, can be complications of FAP and may be detected on detailed imaging. CECT plays a major role in the assessment of FAP patients for surgical interventions, in the assessment of postoperative complications, and in the follow-up of new or recurrent disease. Because FAP is a frequent pathology and its complications are vast, the abdominal cavity assessment is critical with the help of CECT.

Management and surveillance

The management of FAP entails prophylactic surgeries such as colectomy or proctocolectomy to lessen the chances of developing CRC. The kind of surgery that is done depends on the stage of the disease, whether the patient is symptomatic or asymptomatic, and the choice of the patient. Patients need to be followed up for the rest of their lives regarding the remaining rectum or ileal pouch and for the development of extracolonic manifestations.

The management of FAP is mostly in a preventive manner, and some of the interventions are surgical while others are non-surgical. Surgical procedures, which are regarded as the primary therapy, include total colectomy with ileorectal anastomosis and proctocolectomy with ileal pouch-anal anastomosis, decreasing the risk of CRC. Endoscopic examination and polypectomy are other treatments that are non-surgical, but these are not curative.

Sibling A had extensive polyposis with large polyps in the rectum and descending colon; thus, he underwent proctocolectomy with the formation of a J pouch and ileostomy. The above approach is well supported by Hashimoto et al. This is because they note that the above surgeries help reduce the risk of CRC in FAP patients [4]. Sibling B, who also had extensive polyposis with regional lymphadenopathy, was advised to undergo prophylactic colectomy with ileal pouch-anal anastomosis; this is a strategy geared towards reducing the risk of malignancy while preserving bowel function [3,5].

New clinical trials were done on the effectiveness of chemoprevention of familial adenomatous polyposis, which revealed medications such as non-steroidal anti-inflammatory drugs (NSAIDs) have been shown to reduce polyp burden but do not eliminate the need for surgery [13]. The use of low-dose aspirin, for instance, examined the combination of low-dose aspirin and mesalazine, and it was found that aspirin significantly reduced the recurrence of colorectal polyps in Japanese patients with FAP, suggesting its utility as a preventive measure against colorectal cancer. Sulindac and celecoxib are drugs that have been shown to help in reducing the number of adenomatous polyps, thus potentially delaying the need for colectomy or reducing the cancer risk in retained segments of the colon after surgery.

Follow-up and post-treatment challenges

After treatment, patients require lifelong surveillance due to the risk of developing polyps and cancer in the remaining gastrointestinal tract. Challenges post-treatment can include changes in bowel habits, bowel incontinence, and the psychological impact of living with a chronic condition. In most cases, genetic counseling is advised to the patient and his or her family on the implications of the disease and the risk factors that are likely to be inherited in the next generation.

Genetic counseling and next steps

Patients with detected APC gene mutations are encouraged to seek genetic counseling in order to understand the consequences of the disease, the fact that the disease is inherited in an autosomal dominant manner, and the need for screening first-degree relatives [14]. It is advisable to engage in surveillance endoscopy and possibly prophylactic surgery for those who have the mutation. The psychological effect of the disease on patients, particularly young adults and children, should also be taken into account. Such questions as changes in body image after surgery, reproductive choices, and social relationships continue to demand assistance from genetic counselors and other healthcare professionals.

Differential diagnoses

FAP is an autosomal dominant disease causing the production of hundreds to thousands of adenomatous polyps in the colon and rectum during the second decade of life; if untreated, it results in colorectal cancer. Differential diagnosis of FAP is crucial because the management and prognosis can vary significantly among the conditions that mimic its presentation [15]. Differentiating FAP from its mimics primarily involves a combination of clinical evaluation, family history, endoscopic examination, histopathological analysis of the polyps, and genetic testing. Genetic testing is the gold standard for distinguishing between these syndromes, as most have a known genetic basis.

Below are the primary conditions that can mimic FAP and strategies for differentiation:

MUTYH-Associated Polyposis (MAP)

MAP is an autosomal recessive condition characterized by the development of multiple adenomatous polyps and an increased risk of colorectal cancer. Unlike FAP, which is caused by mutations in the APC gene, MAP results from biallelic mutations in the MUTYH gene. Genetic testing for MUTYH mutations can distinguish MAP from FAP. Additionally, patients with MAP tend to have fewer polyps (usually less than 100) compared to those with FAP.

Peutz-Jeghers Syndrome (PJS)

Peutz-Jegers syndrome (PJS) is an autosomal dominant disorder characterized by the development of hamartomatous polyps throughout the gastrointestinal tract and distinctive mucocutaneous pigmentation. The presence of melanin spots on the lips, buccal mucosa, and digits, along with family history and the histological findings of hamartomatous polyps, can help distinguish PJS from FAP. Genetic testing for STK11/LKB1 gene mutations is confirmatory.

Juvenile Polyposis Syndrome (JPS)

Juvenile polyposis syndrome (JPS) is an autosomal dominant condition marked by the presence of multiple juvenile polyps in the colon, stomach, and sometimes in the small intestine [15]. It is differentiated from FAP based on the clinical criteria (presence of more than five juvenile polyps in the colon or multiple juvenile polyps throughout the GI tract) and genetic testing for BMPR1A and SMAD4 mutations, which are absent in FAP.

Lynch Syndrome (Hereditary Nonpolyposis Colorectal Cancer (HNPCC))

Lynch syndrome is an autosomal dominant condition associated with a high risk of colorectal cancer and other cancers but not typically associated with a large number of adenomatous polyps, as in FAP [16]. The absence of a large number of adenomatous polyps and the presence of other Lynch syndrome-associated cancers (endometrial, ovarian, gastric, and others) can suggest Lynch syndrome over familial adenomatous polyposis (FAP). Genetic testing for mismatch repair (MMR) genes (MLH1, MSH2, MSH6, PMS2) mutations is definitive.

Serrated Polyposis Syndrome (SPS)

Serrated polyposis syndrome (SPS) is characterized by multiple serrated polyps in the colon, with an increased risk of colorectal cancer [17]. The histological appearance of serrated polyps, the absence of APC gene mutations, and adherence to the WHO criteria for SPS can help differentiate it from familial adenomatous polyposis (FAP).

**Table 1:** A detailed comparative overview of research on familial adenomatous polyposis: study focus, principal findings, and implications FAP: familial adenomatous polyposis; CHRPE: congenital hypertrophy of the retinal pigment epithelium; GI: gastrointestinal; NSAIDs: non-steroidal anti-inflammatory drugs

Study Reference	Focus of Study	Main Findings	Implications
Half et al., 2009 [1]	General overview of FAP	Provided a comprehensive overview of FAP emphasizing the need for early diagnosis and management.	Highlights the critical role of genetic testing and surveillance in managing FAP.
Leoz et al., 2015 [2]	Genetic basis of FAP	Discussed the genetic mutations in the APC gene and their clinical implications for risk management.	Stressed the importance of personalized medicine approaches in FAP management.
Grover et al., 2012 [3]	Prevalence and phenotypes of APC and MUTYH mutations	Examined the correlation between genetic mutations and the phenotypic presentation of adenomas.	Aided in refining the diagnostic criteria and surveillance strategies for FAP.
Hashimoto et al., 2015 [4]	Common genetic features in adenomas	Compared the genetic features of familial and sporadic adenomas in the upper GI tract.	Provided insights into the molecular pathogenesis common to both types of adenomas.
Groen et al., 2008 [5]	Extra-intestinal manifestations of FAP	Detailed various extra-intestinal manifestations and their management strategies.	Emphasized a holistic approach to managing FAP beyond the colorectal focus.
Devezas et al., 2018 [6]	Case study on large desmoid tumors	Documented successful management strategies for large desmoid tumors in FAP patients.	Underlined the challenges and potential treatment approaches for desmoid tumors in FAP.
Heiskanen et al., 1996 [7]	Treatment results for desmoid tumors	Reviewed the outcomes of different treatment strategies for desmoid tumors in FAP patients.	Offered evidence-based recommendations for treating desmoid tumors.
Traboulsi et al., 1987 [8]	Prevalence of CHRPE in Gardner's syndrome	Explored the occurrence of CHRPE in patients with Gardner's syndrome, a variant of FAP.	Provided diagnostic markers that aid in the early detection of FAP.
Giardiello et al., 1993 [9]	Thyroid and pancreatic cancer risks in FAP	Investigated the increased incidence of thyroid and pancreatic cancers in FAP patients.	Suggested the need for broader cancer screening protocols in FAP.
Giardiello et al., 2014 [10]	Lynch syndrome management guidelines	Established guidelines for the genetic and clinical management of Lynch syndrome.	Assisted in differential diagnosis and management between FAP and Lynch syndrome.
Özsunar et al., 2009 [11]	Efficacy of CT colonography	Evaluated the diagnostic accuracy and patient tolerance for CT colonography in at-risk patients.	Supported the use of alternative imaging modalities for comprehensive FAP assessment.
Panjwani et al., 2011 [12]	Overview of Gardner's syndrome	Provided a detailed review of Gardner's syndrome discussing its clinical and radiologic features.	Helped in understanding the overlap and distinctions between FAP and Gardner's syndrome.
Messersmith et al., 2003 [13]	Advances in pharmacological treatments for colorectal cancer	Reviewed recent pharmacological advances including the role of NSAIDs in colorectal cancer prevention.	Highlighted potential non-surgical interventions that could complement traditional treatments.
Fernández-Suárez et al., 2005 [14]	Genetic counseling in FAP	Discussed the clinical and ethical implications of genetic counseling in managing FAP.	Emphasized the importance of comprehensive genetic education and family screening.
Cichy et al., 2014 [15]	Juvenile polyposis syndrome	Detailed the clinical presentation and management of juvenile polyposis syndrome, differentiating it from FAP.	Informed differential diagnosis strategies in young patients presenting with polyposis.
Cox et al., 2018 [16]	Imaging review of Lynch syndrome	Provided an update on the genomic and imaging aspects of Lynch syndrome.	Assisted in distinguishing Lynch syndrome from FAP through imaging features.
Guarinós et al., 2012 [17]	Serrated polyposis syndrome	Discussed the molecular and pathological aspects of serrated polyposis syndrome.	Offered criteria for differentiating it from FAP in clinical practice.

## Conclusions

The case series highlights the complex nature of familial adenomatous polyposis (FAP) and the importance of genetic markers, imaging, and comprehensive management. Advanced diagnostics and a multidisciplinary approach are essential for effective FAP management. Prophylactic surgeries, continuous surveillance, and chemoprevention with NSAIDs play critical roles in reducing colorectal cancer risk and managing extra-colonic manifestations. Personalized treatment plans and ongoing follow-up are crucial for improving patient outcomes. The review emphasizes the need for ongoing research and innovation to enhance the quality of life and prognosis for individuals affected by FAP.
